# Image-based yield prediction for tall fescue using random forests and convolutional neural networks

**DOI:** 10.3389/fpls.2025.1549099

**Published:** 2025-03-12

**Authors:** Sarah Ghysels, Bernard De Baets, Dirk Reheul, Steven Maenhout

**Affiliations:** ^1^ Department of Plants and Crops, Faculty of Bioscience Engineering, Ghent University, Ghent, Belgium; ^2^ Department of Data Analysis and Mathematical Modelling, Faculty of Bioscience Engineering, Ghent University, Ghent, Belgium

**Keywords:** high-throughput phenotyping, dry matter yield, convolutional neural network, random forest, UAV

## Abstract

In the early stages of selection, many plant breeding programmes still rely on visual evaluations of traits by experienced breeders. While this approach has proven to be effective, it requires considerable time, labour and expertise. Moreover, its subjective nature makes it difficult to reproduce and compare evaluations. The field of automated high-throughput phenotyping aims to resolve these issues. A widely adopted strategy uses drone images processed by machine learning algorithms to characterise phenotypes. This approach was used in the present study to assess the dry matter yield of tall fescue and its accuracy was compared to that of the breeder’s evaluations, using field measurements as ground truth. RGB images of tall fescue individuals were processed by two types of predictive models: a random forest and convolutional neural network. In addition to computing dry matter yield, the two methods were applied to identify the top 10% highest-yielding plants and predict the breeder’s score. The convolutional neural network outperformed the random forest method and exceeded the predictive power of the breeder’s eye. It predicted dry matter yield with an R² of 0.62, which surpassed the accuracy of the breeder’s score by 8 percentage points. Additionally, the algorithm demonstrated strong performance in identifying top-performing plants and estimating the breeder’s score, achieving balanced accuracies of 0.81 and 0.74, respectively. These findings indicate that the tested automated phenotyping approach could not only offer improvements in cost, time efficiency and objectivity, but also enhance selection accuracy. As a result, this technique has the potential to increase overall breeding efficiency, accelerate genetic progress, and shorten the time to market. To conclude, phenotyping by means of RGB-based machine learning models provides a reliable alternative or addition to the visual evaluation of selection candidates in a tall fescue breeding programme.

## 1 Introduction

Plant breeding has made a substantial contribution to global agriculture throughout history. Higher crop yields, resistance to stress factors and enhanced quality traits are but a few examples of its many achievements. Today, the disruptive impact of climate change requires the rapid development of resilient plant varieties. To meet this demand, the field of plant breeding continuously advances, with a key area of innovation being automated high-throughput phenotyping.

Manual plant phenotyping is a labour- and time-intensive endeavour. Moreover, the subjective nature of the process complicates the reproduction and comparison of evaluations (Kumar and Bhatia, 2014). Automated high-throughput phenotyping offers an alternative approach that can address these shortcomings. Generally, this method involves the use of (semi-)autonomous platforms equipped with non-destructive sensors to collect data (Gill et al., 2022). This information is then processed and correlated with phenotypic traits using various data analysis tools, allowing for a fast, large-scale and accurate assessment of traits (Kumar and Bhatia, 2014; Awada et al., 2018). However, because the mathematical relationship between sensor data and the trait of interest is often unknown, an analysis method is required that can autonomously establish this connection for large datasets. As a result, machine learning techniques have become increasingly popular in recent years (Lin et al., 2019; Atieno et al., 2017; Ramcharan et al., 2019; Sandhu et al., 2021).

Machine learning was first defined by Arthur L. Samuel in 1959 as ‘a field of study that gives computers the ability to learn without being explicitly programmed’. Within the scope of automated phenotyping, this definition implies that an algorithm can learn the relation between the collected sensor data and the trait of interest without additional guidance from the breeder. This study explores the potential of two machine learning techniques: Random Forest (RF) and Convolutional Neural Network (CNN). RF is a method developed by Breiman in 2001. The algorithm aggregates multiple decision trees to form one powerful, robust prediction model. RFs have become widely popular due to their versatility, ease-of-use and high prediction accuracy (Biau and Scornet, 2016; Belgiu and Drăguț, 2016; James et al., 2023). Additionally, the approach distinguishes itself from many other machine learning techniques by providing straightforward methods to determine feature importance, offering valuable insight into an otherwise opaque modelling process. These advantages motivated our selection of RF for this study. However, when applied to visual data analysis, RFs require the manual extraction of informative predictors, or ‘features’, from images, as they cannot process raw pixel data directly. Selecting the optimal feature extraction method for every task is a time-consuming process that requires a high level of expertise. A breakthrough in this area was the development of CNNs, which can extract features autonomously. CNNs are a type of neural network particularly suited for image analysis (Loussaief and Abdelkrim, 2018). The algorithm detects spatial patterns in images via specialised operations within its ‘convolutional layers’ (Tuba et al., 2021) and uses these patterns as features in the prediction process. CNNs are currently considered the state-of-the-art technology in various computer vision applications, *if* they are provided with sufficient, high-quality training data.

In this study, we propose RF and CNN models to analyse RGB images captured by an unmanned aerial vehicle (UAV or ‘drone’). The combination of RGB imaging and UAV technology was chosen to facilitate practical application, ensuring data collection is simple, fast and cost-effective. The dry matter yield (DMY) of individual tall fescue (TF) plants is the phenotypic trait of interest. TF [*Festuca arundinacea (Schreb.)*] was selected due to its notable resilience to the effects of climate change in Northwestern Europe. The grass species is known to be tolerant to both drought and flooding, in contrast to perennial ryegrass, which is currently the most popular pasture grass in these regions (Gibson and Newman, 2001; Reheul, 2021; Mosimann et al., 2010; Cougnon et al., 2014). In addition to predicting DMY, we aim to identify the highest-yielding plants. Although DMY is a very interesting trait, breeders do not necessarily need to know the exact yield of a plant to consider it for selection. Essentially, they only require an efficient way to determine whether a plant belongs to the top-performing group and should advance to subsequent breeding phases or if it should be discarded. This ‘Top-performers problem’ constitutes our second research question. Finally, we explore the prediction of the breeder’s scores as a third objective of our research. Although the breeder explicitly evaluated DMY in this study, these observations could still be biased by other traits, such as disease or deficiency symptoms. Therefore, it was tested whether a model could capture these additional visual fitness characteristics as well.

To date, few published studies have focussed on estimating yield in forage species using RGB images. Notable examples include the work of Castro et al. (2020); de Oliveira et al. (2021) and de Souza Rodrigues et al. (2023), who applied CNNs to estimate yield in various genotypes of Guinea grass (*Panicum maximum*). Similarly, Oliveira et al. (2022) used a CNN to estimate DMY in a Timothy-meadow fescue mixture. While these studies provided valuable insights and achieved commendable prediction accuracy, they had certain limitations. Firstly, they used relatively small datasets, with the first three studies analysing only 330 plant plots and the last one just 96. Secondly, they examined plots containing multiple individual plants or pasture sections, where yield was averaged across the plot. Since this approach reduces outliers, the model is not trained to estimate the yield of exceptionally high-performing individuals — plants that are particularly valuable in a breeding programme. Therefore, the present study explores whether a high-throughput phenotyping approach could accurately estimate the yield of these top-performing individuals, using a fairly large dataset of 4,224 plots. Furthermore, to the best of our knowledge, no previous research includes the visual assessment of a breeder into the analysis. This is a valuable addition, however, as it allows for a direct comparison between the accuracy of the automated phenotyping approach and the manual phenotyping method, providing valuable insight into the potential improvement in selection accuracy if the tested method were implemented in a breeding programme.

Another popular method to predict biomass yield involves plant height models (Borra-Serrano et al., 2019; Grüner et al., 2019; Fu et al., 2021). While this approach allows for reliable estimates, the additional requirements with respect to data collection and processing somewhat increase the adoption threshold (Castro et al., 2020). Therefore, this study proposes the use of standard RGB images, analysed using machine learning methods, as a more accessible phenotyping solution.

## 2 Materials and methods

### 2.1 Study area and data acquisition

#### 2.1.1 Field trial

**Figure 1:**
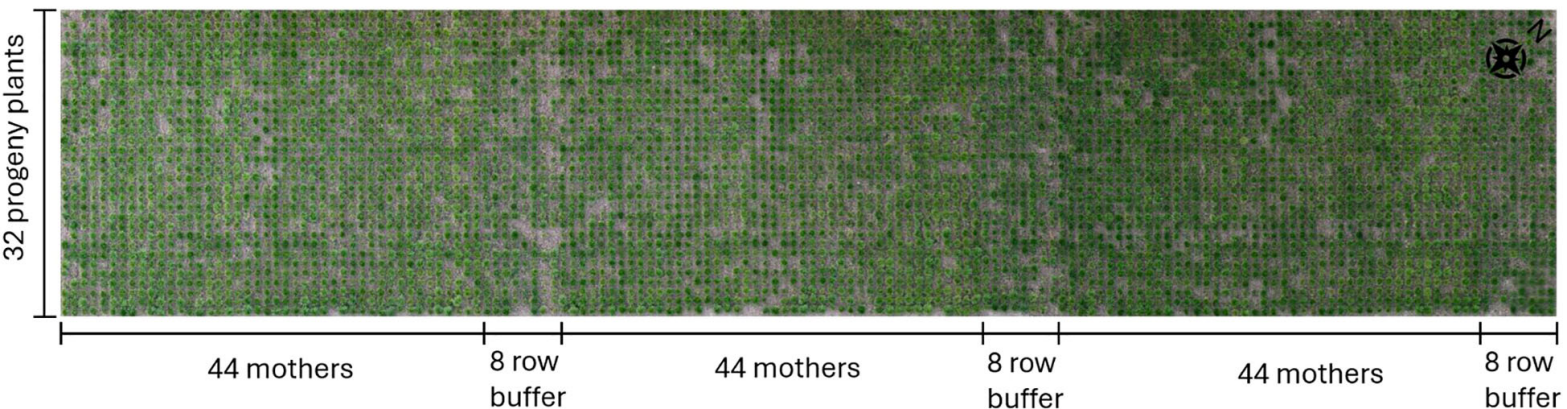
Organisation of the studied TF field.

The tall fescue (TF) field was located at Proefhoevestraat 22 in Melle, Belgium, as part of Ghent University’s TF breeding programme. It was established as a progeny assessment trial, evaluating the breeding value of 44 mothers based on the performance of their 32 half-sib progeny across three replications (see **Figure 1**). The plants were sown in trays on the 20th of August 2019 and transplanted to the field on the 15th of October. Individual plants were spaced 0.5 m apart within and between rows. The data were collected in the spring of 2022, marking the third year of field evaluation for the progeny.

#### 2.1.2 Image acquisition

RGB images were captured using a DJI Matrice 200 UAV, mounted with a Zenmuse X5S camera. The flight took place on the 11th of April 2022 at a height of 40m, resulting in a resolution of 0.91 cm/pixel, with a frontal and lateral image overlap of 80%. The orthoimage was generated using the open source software OpenDroneMap, version 2.8.4. Subsequently, the field was divided into individual plots for each plant. This segmentation was initially performed using R functions from the FIELDimageR package (Matias et al., 2020). However, since the layout of the progeny field did not fully adhere to a rectangular grid, some clips were imperfect, which we assumed would impair model accuracy. Therefore, the images were re-segmented by visually selecting the optimal clip location per plot, instead of relying on the locations of the grid, using a custom Python script.

#### 2.1.3 *In-situ* data collection

The plants were individually scored by an experienced TF breeder on the 11th of April, 2022. The original scores ranged from zero to five, with zero indicating the worst performance and five the best. The assessment is primarily an estimation of biomass, based on proxy traits such as plant volume and tiller density. However, other relevant factors, such as the presence of disease symptoms, likely influenced the evaluation process. These scores were aggregated into three classes: Class 1 for scores of four and five, Class 2 for a score of three, and Class 3 for scores of two, one and zero. This classification aims to simplify the practical use of the model’s outcomes by plant breeders, as interpreting and implementing categories of ‘good’, ‘medium’ and ‘bad’ performers is more intuitive than using five distinct classes. From the 12th to the 14th of April, the plants were harvested using a hedge trimmer whose blade slid across the top of a 30cm square frame with a height of 5cm. The collected biomass of each plant was weighed, dried for three days at 70°C and then reweighed to determine the DMY.

### 2.2 Data exploration

**Figure 2:**
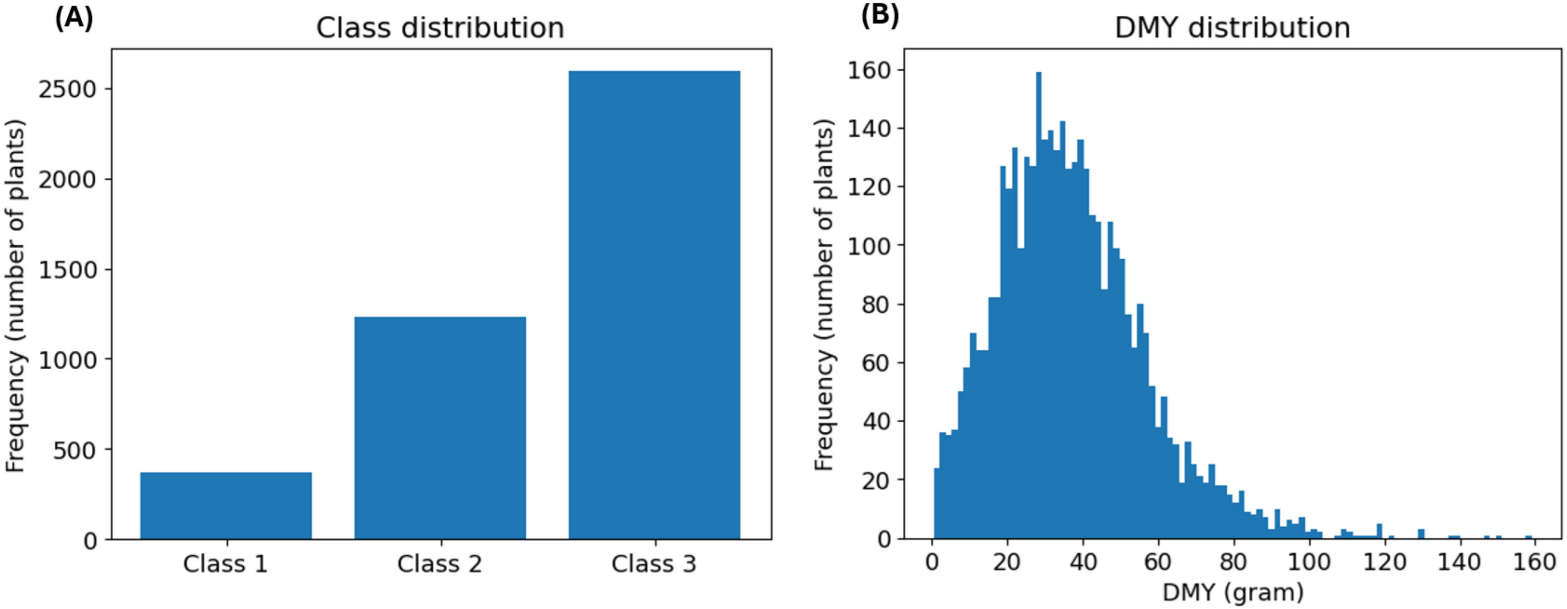
**(A)** depicts the prevalence distribution of the classes based on the breeder’s score. **(B)** illustrates the distribution of the DMY in gram. The histogram consists of 100 bins with a width of 1.6 gram.

First, we examined the distribution of the breeder’s score, shown in **Figure 2A**. This analysis revealed a notable imbalance: Class 1 was the least prevalent, while Classes 2 and 3 were moderately and highly represented, respectively. This distribution was expected, since Classes 2 and 3 contain the most common, average scores (2 and 3). Additionally, Class 3 contains the highest number of different scores (0, 1 and 2), further explaining its large size. This class imbalance was addressed in both model design and evaluation. Next, we assessed the distribution of the DMY, which was slightly right-skewed, as depicted in **Figure 2B**. The extreme observations in the right tail of the distribution might complicate prediction. However, there is no reason to assume that these measurements are incorrect and the highest yields are the most interesting for a breeder. Therefore, no suspected outliers were removed from the dataset.

### 2.3 Datasets

Before training the models, 10% of the data was reserved as a stratified test set to evaluate the performance of the final, optimised models. The remaining 90% was split into a training set (80%) and a validation set (20%) for model training and hyperparameter optimisation. To ensure robust evaluation, three stratified train-validation splits were performed, allowing each tuning configuration to be tested three times.

### 2.4 Models

We explored three different problems: estimating DMY, identifying top-performing individuals and predicting the breeder’s score. For the first problem, we developed a regression model that uses RGB images as explanatory variables to estimate the DMY of individual TF plants. This model will be referred to as the ‘DMY model’. Additionally, we wanted to compare the predictive power of this image-based model to the ability of the breeder to estimate DMY. Therefore, a linear model was added that uses the breeder’s score as a predictor for DMY. This comparative model is called the ‘Benchmark DMY model’.

The second problem focuses on the identification of the top-performing individuals. While a ranking algorithm initially seems the most suitable approach, we opted to use binary classification. This method provides a better fit to the requirements of a plant breeder, who’s primary objective is to distinguish high-yielding plants that should be used in subsequent breeding stages from lower-performing individuals that should be discarded. The exact ranking of the individuals within these ‘Select’ and ‘Discard’ groups is of marginal importance. Therefore, a binary classification model was developed, with the ‘Select’ class containing the top 10% highest-yielding individuals and the ‘Discard’ class comprising the remaining 90%. This threshold can be adjusted to align with the objectives and budget of the breeder. This model is referred to as the ‘Top-performers model’.

Lastly, we created a model to predict the breeder’s score. With this approach we aimed to not only capture the DMY but also detect other visual characteristics that influence a plant’s value. As the breeder’s score is an ordinal variable, ordinal regression would be the most appropriate analytical approach. However, ordinal regression is used less frequently and is therefore less straightforward to implement. It requires more manual coding and consequently more time and expertise. Hence, we initially opted for a classification model and evaluated the suitability of this simplification in the Discussion section. This model is called the ‘Breeder’s score model’.

**Figure 3:**
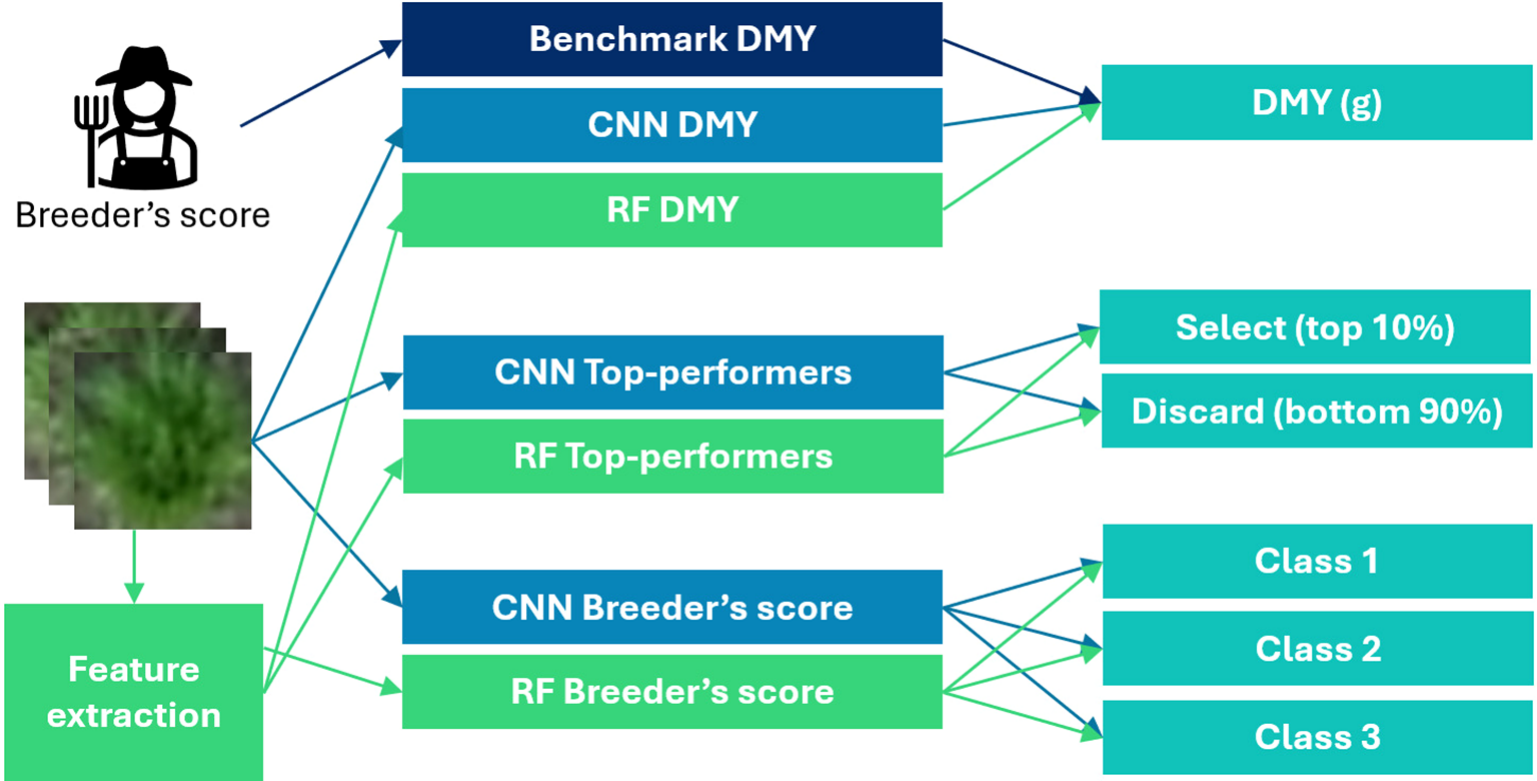
Schematic overview of the seven models created in this study.

These three problems were modelled in both the RF and CNN framework. **Figure 3** provides an overview of the seven resulting models.

### 2.5 Benchmark model

The breeder-assigned scores were used as categorical predictors in a linear model for DMY (Equation 1):


(1) $$\text{DMY}=\beta_{0}S_{0}+\beta_{1}S_{1}+\beta_{2}S_{2}+\beta_{3}S_{3}+\beta_{4}S_{4}+\beta_{5}S_{5}$$

Here, *β*_0_ through *β*_5_ are the coefficients representing the average yield corresponding to plants with scores 0 through 5, respectively. The binary variables *S*_0_, *S*_1_,…, *S*_5_ indicate the presence of each score. For instance, *S*_0_ = 1 signifies that the plant has a score of 0, while *S*_1_ = 1 indicates a score of 1, and so on. Only one of these binary variables will be 1 for any given observation, since scores are mutually exclusive. The model uses the original scoring scale (0 to 5), as the additional level of detail provides extra information when used as predictors. Conversely, when the breeder’s score serves as the response variable in the ‘Breeder’s score’ model, the specific distinctions between individual scores are less important. Instead, the focus shifts to classifying plants into broader categories: good plants (Class 1) versus mediocre (Class 2) and poor plants (Class 3).

The accuracy of this linear model serves as an assessment of the predictive power of the breeder’s visual evaluation of selection candidates. It is compared to the accuracy of the image-based DMY models to evaluate whether the automated phenotyping approach provides an improvement over the manual method and to quantify this potential enhancement.

### 2.6 Random forest

Random Forest (RF) is a machine learning algorithm that combines a large number of randomised decision trees to solve a classification or regression problem (Breiman, 2001). Decision trees divide observations into groups based on their characteristics or features. The objective is to create segments where the response variables are predominantly of the same class or have comparable numerical values (James et al., 2023). The quality of each division, or ‘split’, is determined by a splitting criterion, which quantifies the similarity of the observations in the resulting groups, or ‘nodes’. The algorithm iteratively finds the feature and value that create the best split, continuing until the nodes are sufficiently homogeneous. Once the tree is constructed, predictions can be made by assigning a new observation to a node based on its features. The predicted outcome is then determined as the dominant class in that node for classification tasks or the average value for regression tasks. While decision trees achieve high prediction accuracy on the training data, they usually do not generalise well to new data. RF addresses this limitation by averaging outcomes from many decision trees, improving their predictive power. More detailed information on RFs can be found in Breiman (2001); Biau and Scornet (2016) and James et al. (2023).

#### 2.6.1 Feature extraction

Since RF models cannot process raw image data directly, informative features have to be extracted. This study uses a colour histogram (Chapelle et al., 1999; Cheng and Chen, 2003), Hu moments (Hu, 1962), Haralick features (Haralick et al., 1973), green pixel counts and vegetation indices. The first three methods aim to attain a broad overview of the images’ colour, shape and texture, following the work of Nakhle and Harfouche (2021). The number of green pixels was added as an intuitive estimate of plant coverage. First, the pixels that matched a variety of predefined green shades were counted. This approach resulted in a slight underestimation of the true number of plant pixels. To address this issue, we developed a second method that described green pixels more broadly, based on the ratio of the green band to the red and blue bands. This more general definition overestimated the number of plant pixels. The combination of the two features was assumed to give a fairly accurate approximation. Lastly, four RGB-based vegetation indices were chosen, inspired by similar studies (Lussem et al., 2018; Li et al., 2016). An overview of their specifications is presented in **Table 1**.

**Table 1:** Overview of chosen vegetation indices.

Name	Expression	Reference
Red-Green-Blue Vegetation Index	$\text{RGBVI}=\frac{\left(G\cdot G\right)-\left(R\cdot B\right)}{\left(G\cdot G\right)+\left(R\cdot B\right)}$	(Bendig et al., 2015)
Green Leaf Index	$\text{GLI}=\frac{2\cdot G-R-B}{2\cdot G+R+B}$	(Louhaichi et al., 2001)
Visible Atmospherically Resistant Index	$\text{VARI}=\frac{G-R}{G+R-B}$	(Gitelson et al., 2002)
Normalised Green Red Difference Index	$\text{NGRDI}=\frac{G-R}{G+R}$	(Gitelson et al., 2002)

*R*, *G* and *B* represent the intensities of the red, green and blue channels, respectively.

#### 2.6.2 Model optimisation and analysis

When training the RF models, two hyperparameters were fixed and the importance of four hyperparameters was evaluated using a grid search. The two fixed hyperparameters included the number of trees and the maximum number of features the trees could select from to make each split. The number of trees was set to 1000, following an informal exploration to balance computational efficiency with model performance. The maximum number of features considered per split was defined according to standard guidelines (James et al., 2023; Probst et al., 2019) as the square root of the total number of features for classification and one-third of the features for regression.

The four formally optimised hyperparameters were: the use of balanced class weights (yes/no), the splitting criterion (Gini impurity/Entropy for the two classification models and Mean squared error/Friedman mean squared error/Mean absolute error/Poisson deviance for the DMY model), maximum tree depth (3/5/7) and the complexity parameter (alpha) for cost-complexity pruning (ten evenly spaced values between 10^−3^ and 1). The use of balanced class weights was evaluated to address the imbalance in the Top-performers and Breeder’s score datasets. Each class is assigned a weight inversely proportional to its size, to ensure equal contribution to the splitting criterion. Different splitting criteria were tested to explore alternative methods of purity quantification in this context, while the last two hyperparameters were used to minimise overfitting. These hyperparameters were optimised using a cross-validated grid search. In this procedure, a range of values for each hyperparameter is given and the algorithm tests every possible combination using cross-validation. The best performing configuration is reported as the set of optimal hyperparameter values, which were used when evaluating the models on the test set.

The Entropy (E) and Mean Squared Error (MSE) were the most influential splitting criteria in this study. Therefore, their equations for node *m* are given in Equations 2, 3:


(2) $${\text{E}}_{m}=-\sum_{i=1}^{C}\;p_{i}\;\text{log }p_{i},$$

with *C* the number of classes and $p_{i}$ the proportion of class *i* observations in node *m*.


(3) $${\text{MSE}}_{m}=\frac{1}{N}\sum_{i=1}^{N}{\left(y_{i}-\bar{y}\right)}^{2},$$

where *N* represents the number of samples in node *m*, $y_{i}$ the DMY of observation i of node m and $\bar{y}$ the mean DMY in node *m*.

Following model optimisation, feature importance was determined to assess the impact of each extracted feature. The importance was measured by calculating how much of the total reduction in the splitting criterion was achieved due to splits involving that particular feature, with a measure called Gini Importance or Mean Decrease in Impurity. These values signify the impact of each feature relative to the others, providing insight into the model’s prediction process. Subsequently, feature selection was carried out, which evaluates the importance of each feature and removes those with an importance below a certain threshold. The threshold for retaining features was first set to the mean of all feature importances and later to a stricter threshold of 0.01, meaning 1% of the combined Gini importance of all features. For both thresholds the performance and required computation time of the RF models were evaluated.

### 2.7 Convolutional neural network

A Convolutional Neural Network (CNN) is a type of neural network that is well suited to analyse image data (James et al., 2023). Neural networks are nonlinear statistical models that are, as the name suggests, loosely based on the structure of interconnected neurons in the brain. Mimicking the behaviour of its biological counterpart, the artificial neuron accepts signals from various neighbours as input, processes them and either fires a signal or remains inactive based on the result (Zupan, 1994). Not all neighbouring neurons will have the same impact on its activation, which is why they are assigned weights. Furthermore, the ease with which a neuron fires also varies, which is enabled by biases.

All neural network architectures contain layers of neurons, which are all interconnected to the adjacent layers. In addition to these ‘fully-connected layers’, most architectures include specialised layers. An example is the convolutional layer, characteristic to the CNN. It performs a specific type of computation, a ‘convolution’, to assess spatial relations between the input neurons. When the input neurons are pixels of an image, this means the CNN can not only analyse the pixel values but also their spatial context, enabling the model to extract meaningful image features. This automatic feature extraction, coupled with the analytical power of the fully-connected layers, makes the CNN algorithm efficient and potentially very accurate. Further reading on CNNs can, among others, be found at James et al. (2023); Bhatt et al. (2021) and LeCun et al. (2010).

#### 2.7.1 Model optimisation

The CNN models were built using four pre-trained architectures, namely VGG-16 (Simonyan and Zisserman, 2014), Densenet161 (Huang et al., 2017), EfficientNetV2 (Tan and Le, 2021) and ResNet50 (He et al., 2016). VGG-16, while an older model, was included to allow comparisons with previous studies. The other three models were selected for their relatively low parameter counts, while still achieving commendable accuracy. The choice for smaller models was motivated by constraints in computational resources and available data (Pasupa and Sunhem, 2016). The fully connected layers of these architectures were substituted with custom code for each model. More precisely, the DMY model was adapted to output a single value, the Top-performers model two and the Breeder’s score model three.

The DMY model was trained using the MSE loss function (Equation 4):


(4) $$\text{MSE}=\frac{1}{N}\sum_{i=1}^{N}{\left(y_{i}-{\hat{y}}_{i}\right)}^{2},$$

with *N* the number of samples in the training set, $y_{i}$ the measured DMY and ${\hat{y}}_{i}$ the predicted DMY. The Top-performers and Breeder’s score models made use of the Cross-Entropy loss criterion (CE, Equation 5):


(5) $$\text{CE}=-\sum_{i=1}^{N}\sum_{j=1}^{C}y_{ij}\;\text{log }{\hat{p}}_{ij},$$

with *N* the number of samples in the training set, *C* the number of classes, $y_{ij}$ an indicator variable that takes the value 1 if class *j* is the true class of sample *i* and 0 otherwise and ${\hat{p}}_{ij}$ the predicted probability of sample *i* belonging to class *j*. The same loss functions were used for similar approaches in previous studies (Castro et al., 2020; de Oliveira et al., 2021; Semenov et al., 2019). Compensating class weights were applied in the loss function to address the imbalance in the Top-performers and Breeder’s score datasets. If the correct classification of the ‘Select’ class and Class 1 should be prioritised further, higher weights can be given to these classes. However, in the scope of this study, the class weights were selected to ensure that each class contributed equally to the loss function, regardless of size.

The models were trained for 15 epochs, meaning all training data was used 15 times to update the parameters. The results section reports the prediction accuracy of the best-performing epoch. Additionally, it was assessed which pre-trained architecture performed the best and whether using pre-trained model parameters enhanced accuracy.

### 2.8 Model evaluation

The DMY model was evaluated using R^2^ and Root Mean Square Error (RMSE). Since R^2^ is a widely-used, unitless metric, it allows for comparison across different datasets and studies, while RMSE is easily interpretable as it is expressed in the original unit of measurement. Their definition is given in Equations 6, 7, with *N* representing the number of observations, $y_{i}$ the response value of observation *i*, $\bar{y}$ the mean of the response values and ${\hat{y}}_{i}$ the prediction made by the model for observation *i*.


(6) $${\text{R}}^{2}=1-\frac{\sum_{i=1}^{N}{\left(y_{i}-{\hat{y}}_{i}\right)}^{2}}{\sum_{i=1}^{N}{\left(y_{i}-\bar{y}\right)}^{2}}$$

(7) $$\text{RMSE}=\sqrt{\frac{\sum_{i=1}^{N}{\left(y_{i}-{\hat{y}}_{i}\right)}^{2}}{N}}$$

The performance metrics for the two classification models were centred around the confusion matrix. Although this matrix is very informative – it gives a comprehensive overview of all correctly and incorrectly classified observations – it is less convenient when comparing evaluations. Therefore, metrics comprising of a single value were added as well. These measures are based on various combinations of precision (P), recall (R) and specificity (S), which in turn consist of different configurations of the True Positives (TP), True Negatives (TN), False Positives (FP) and False Negatives (FN) of the confusion matrix (Equations 8–10).


(8) $$\text{Precision }\left(\text{P}\right)=\frac{\text{TP}}{\text{TP}+\text{FP}}$$

(9) $$\text{Recall }\left(\text{R}\right)=\frac{\text{TP}}{\text{TP}+\text{FN}}$$

(10) $$\text{Specificity }\left(\text{S}\right)=\frac{\text{TN}}{\text{TN}+\text{FP}}$$

For both the Top-performers and Breeder’s score models, the Balanced Accuracy (BA) was used to account for the imbalanced classes during model evaluation. BA is defined in Equation 11 as the average of the recall for each class *i*, with *C* indicating the total number of classes:


(11) $$\text{Balanced accuracy }\left(\text{BA}\right)=\frac{1}{C}\sum_{i=1}^{C}\;R_{i}$$

Lastly, the F-measure was added as a third metric. The F_1_-measure added a different perspective to the evaluation of the Breeder’s score model (Equation 12) and the F_2_-measure made it possible to favour correctly classifying the top 10% class over the bottom 90% class in the Top-performers model (Equation 13):


(12) $${\text{F}}_{1}=\frac{2\cdot \text{P}\cdot \text{R}}{\text{P}+\text{R}}$$

(13) $${\text{F}}_{2}=\frac{\left(1+2^{2}\right)\cdot \text{P}\cdot \text{R}}{\left(2^{2}\cdot \text{P}\right)+\text{R}}$$

## 3 Results

### 3.1 Predictive power of the breeder’s score

To benchmark the predictive performance of the image-based RF and CNN models, we evaluated the breeder’s ability to predict DMY. A linear model was fitted using the breeder’s score as a categorical predictor. **Table 2** presents this benchmark DMY model’s performance on the training and test sets.

**Table 2:** R^2^ on the train and the test set and RMSE on the test set for the linear model using the breeder’s score as a categorical predictor.

Evaluation metric	Performance
Train set R^2^	0.54
Test set R^2^	0.54
Test set RMSE (g)	14

### 3.2 Feature importance in the random forest models

**Figure 4:**
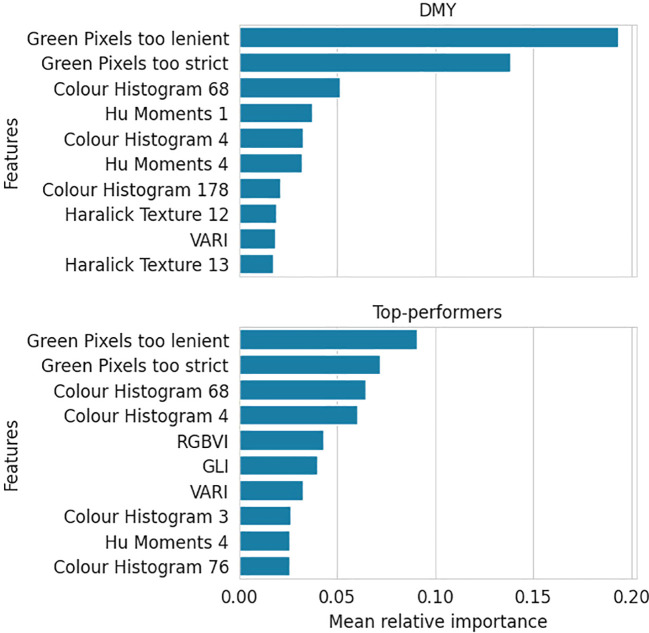
Mean relative importance over three train-validation set splits of the top ten most important features for the DMY and Top-performers RF models.

**Figure 4** illustrates the ten most impactful features for the DMY and Top-performer RF models, along with their relative importances. The DMY model attributes great importance to only a few features, followed by a steep decline. Conversely, the Top-performer model presents a moderately important top feature and shows a gradual descent thereafter.

Furthermore, the figure indicates that the ten most important features are largely the same for the two response types. The two green pixel counters, the 4th and 68th colour histogram features and the VARI vegetation index were important in both models.

**Figure 5:**
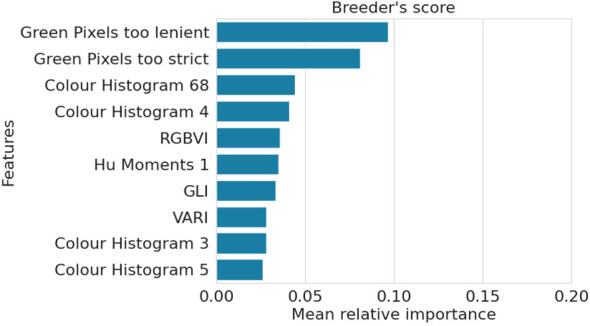
Mean relative importance over three train-validation set splits of the top ten most important features for the Breeder’s score RF model.

The RF model predicting the breeder’s score exhibits an importance distribution similar to that of the Top-performers model (**Figure 5**). Moreover, it shares many of its most important features with the other two models, particularly the Top-performers model, with which it shares 8 of the 10 most impactful features.

Secondly, feature selection was explored for all RF models using two importance thresholds: the mean of all importances, which is less strict and model-dependent, and the absolute threshold of 0.01 (1% of combined Gini importance), which proved to be more stringent. Training the models with these feature subsets resulted in negligible differences in performance and computation time (results not shown).

### 3.3 Pre-trained architectures in the CNN models

**Figure 6:**
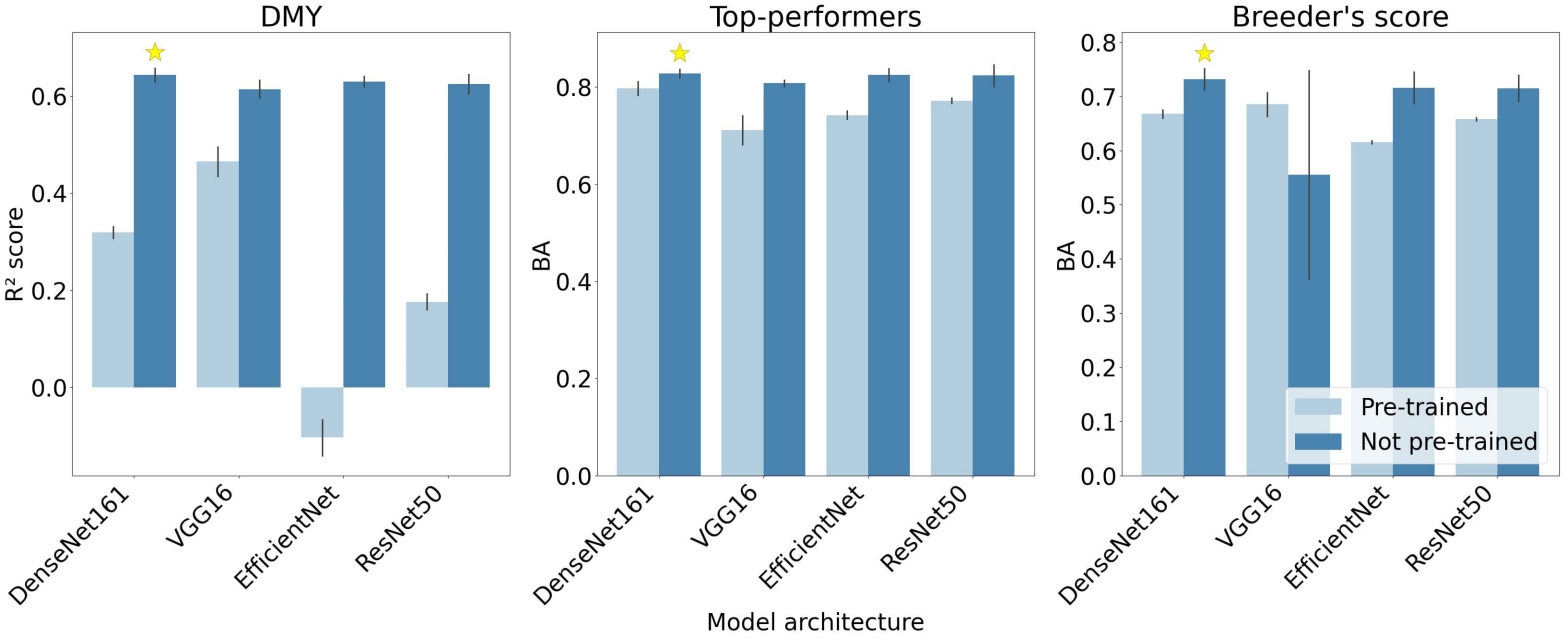
Mean scores of the four CNN architectures across the three train-validation set splits. The highest-performing architecture for each response type is indicated with a star. The size of each errorbar represents two standard deviation units. The depicted performance metrics are the BA and R2 score, the RMSE, F1- and F2-measures followed similar trends (results not shown).

The predictive performance of the four CNN architectures are compared for the DMY and Top-performers models in the left and middle graphs of **Figure 6**. These architectures were tested both with pre-trained weights and biases, where only the parameters of the final layers were updated during training, and without pre-trained parameters. The models with pre-trained parameters demonstrated a lower performance across all architectures and both response types. This performance gap was particularly pronounced in the DMY architectures. Additionally, all architectures performed similarly, with DenseNet161 slightly outperforming the others and VGG-16 minimally lagging behind.

For the Breeder’s score model, all architectures performed better without pre-trained parameters, except for the VGG-16 architecture (right graph in **Figure 6**). The VGG-16 model without pre-trained weights also portrayed a very large standard deviation compared to the other architectures.

The two metrics used to evaluate the performance of the Breeder’s score model did not agree on the best architecture: BA favoured DenseNet161, while the F_1_-measure ranked ResNet50 the highest. To better understand these contrasting results, the diagonal values of the confusion matrices—representing the percentage of correctly classified images per class—for the two architectures are compared in **Table 3**. DenseNet161 owes its higher BA to a higher performance on Classes 1 and 2, whereas ResNet50 scored better for Class 3. Since Class 1 is more important for the breeding process, DenseNet161 was selected as the optimal architecture.

**Table 3:** Mean percentage of correctly classified images per class for both the DenseNet161 and ResNet50 architectures in the CNN Breeder’s score model.

Breeder’s score model	Percentage of correctly classified images		
	Class 1	Class 2	Class 3
DenseNet161, no pre-trained parameters	77	66	76
ResNet50, no pre-trained parameters	71	65	78

The best-performing architecture for each class is indicated in blue.

As these results exemplify, the F_1_-measure was not ideal for the evaluation of the Breeder’s score models. While we expected it to provide an additional perspective alongside BA, it generally differed only slightly. When it did favour a different model, this was primarily due to a stronger emphasis on Class 3 rather than Classes 1 and 2, which was undesirable in this context. A more suitable approach would have been to replace the F_1_-measure with a metric that explicitly prioritises Class 1 above Classes 2 and 3, such as the recall or F_2_-measure for Class 1.

**Figure 7:**
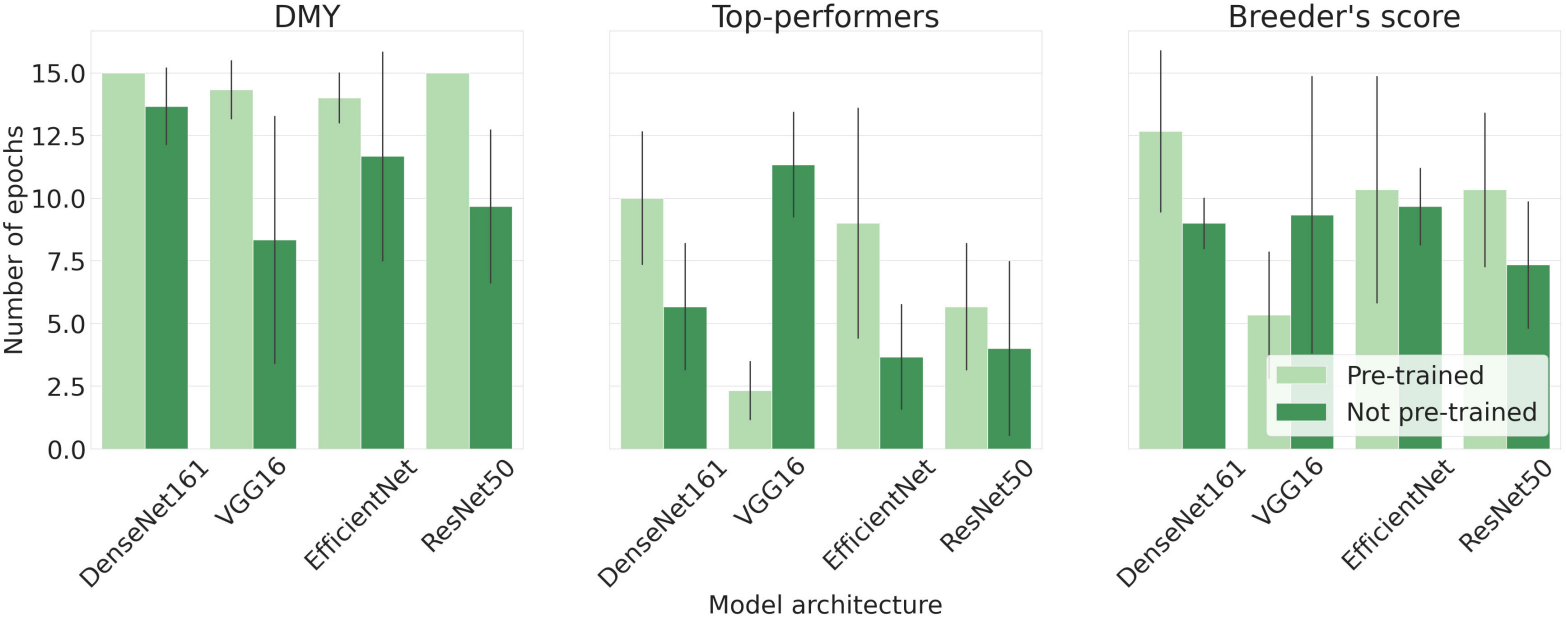
Mean epoch at which the model portrayed its highest performance. The size of each error bar is twice the standard deviation of the mean.

Lastly, **Figure 7** illustrates the mean epoch, serving as an indirect measure of computation time, at which each model architecture reached its peak accuracy. The DMY models required the most epochs to identify the general trend, while the Top-performers models needed the least time. The Breeder’s score model is positioned between these two. For all architectures, except VGG-16, models with pre-trained parameters required more epochs compared to their non-pre-trained counterparts. Moreover, the errorbars shown in **Figure 7** indicate that the number of epochs varied considerably for different train-validation set splits.

### 3.4 Evaluation on the test set

For each model, the hyperparameters and architectures that achieved the highest prediction accuracy in prior analyses were used to assess the performance of the models on the test set. All results are detailed in **Table 4**.

**Table 4:** Hyperparameters and architectures used in the optimised models and the models’ performance on the test set.

	Hyperparameters/Architecture	Performance
RF		
DMY	Default	0.59 (R²) 13g (RMSE)
Top-performers	Entropy, max depth 7, ccp alpha 0.001	0.78 (BA) 0.58 (F_2_)
Breeder’s score	Entropy, max depth 7, ccp alpha 0.001	0.71 (BA) 0.68 (F_1_)
CNN		
DMY	DenseNet161	0.62 (R²) 13g (RMSE)
Top-performers	DenseNet161	0.81 (BA) 0.63 (F_2_)
Breeder’s score	DenseNet161	0.74 (BA) 0.68 (F_1_)

**Figure 8:**
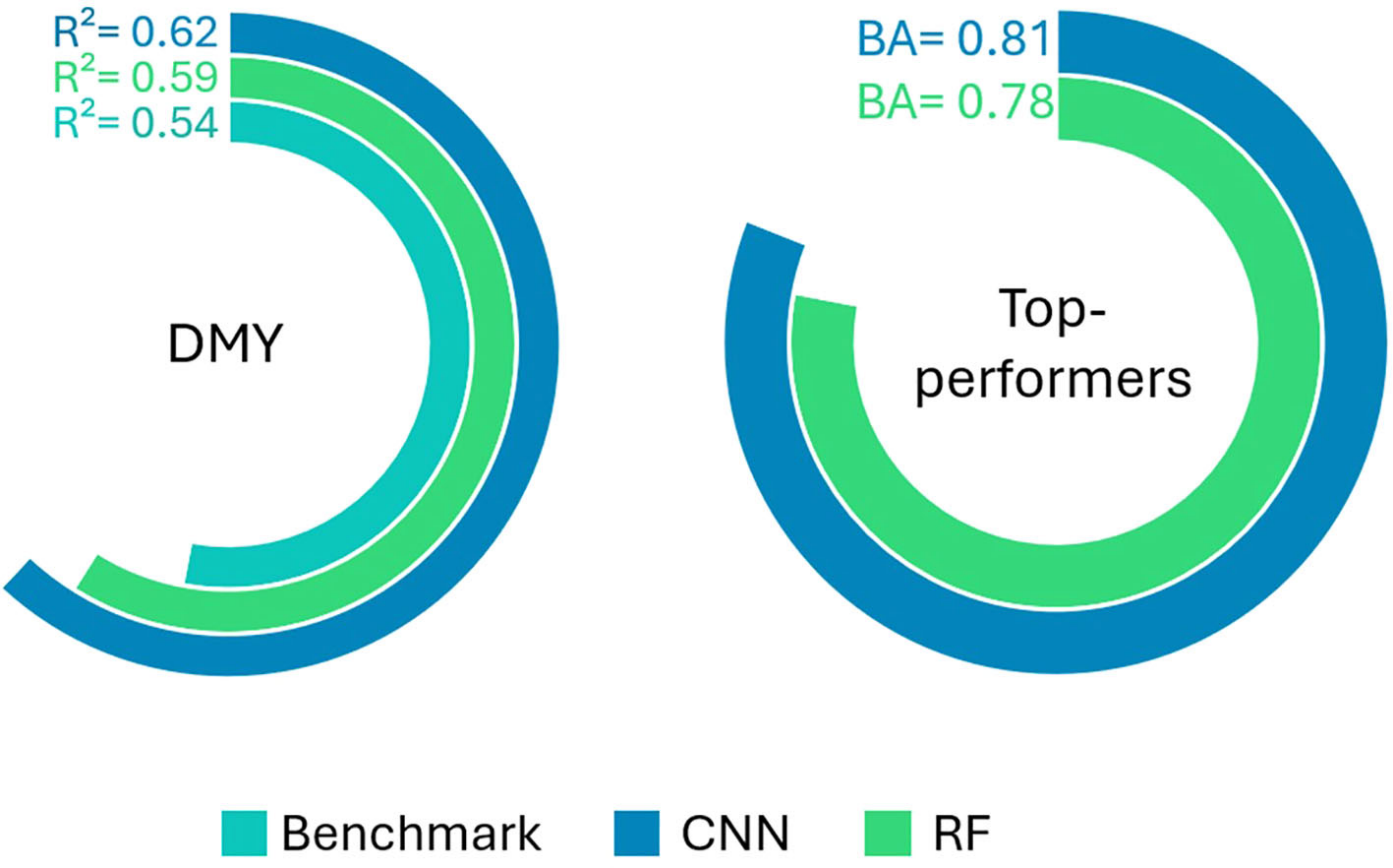
R² score of the Benchmark, RF and CNN DMY models (left panel) and Balanced Accuracy (BA) of the CNN and RF Top-performers models (right panel).

The performance of the DMY and Top-performers models are visually represented in **Figure 8**. The predictive power of the image-based DMY models is also compared to that of the Benchmark DMY model, which uses the breeder’s score as a predictor variable. The CNNs slightly outperformed the RF models for both response types. Furthermore, both the CNN and RF image-based models demonstrated notably better performance in predicting DMY compared to the Benchmark model.

**Figure 9:**
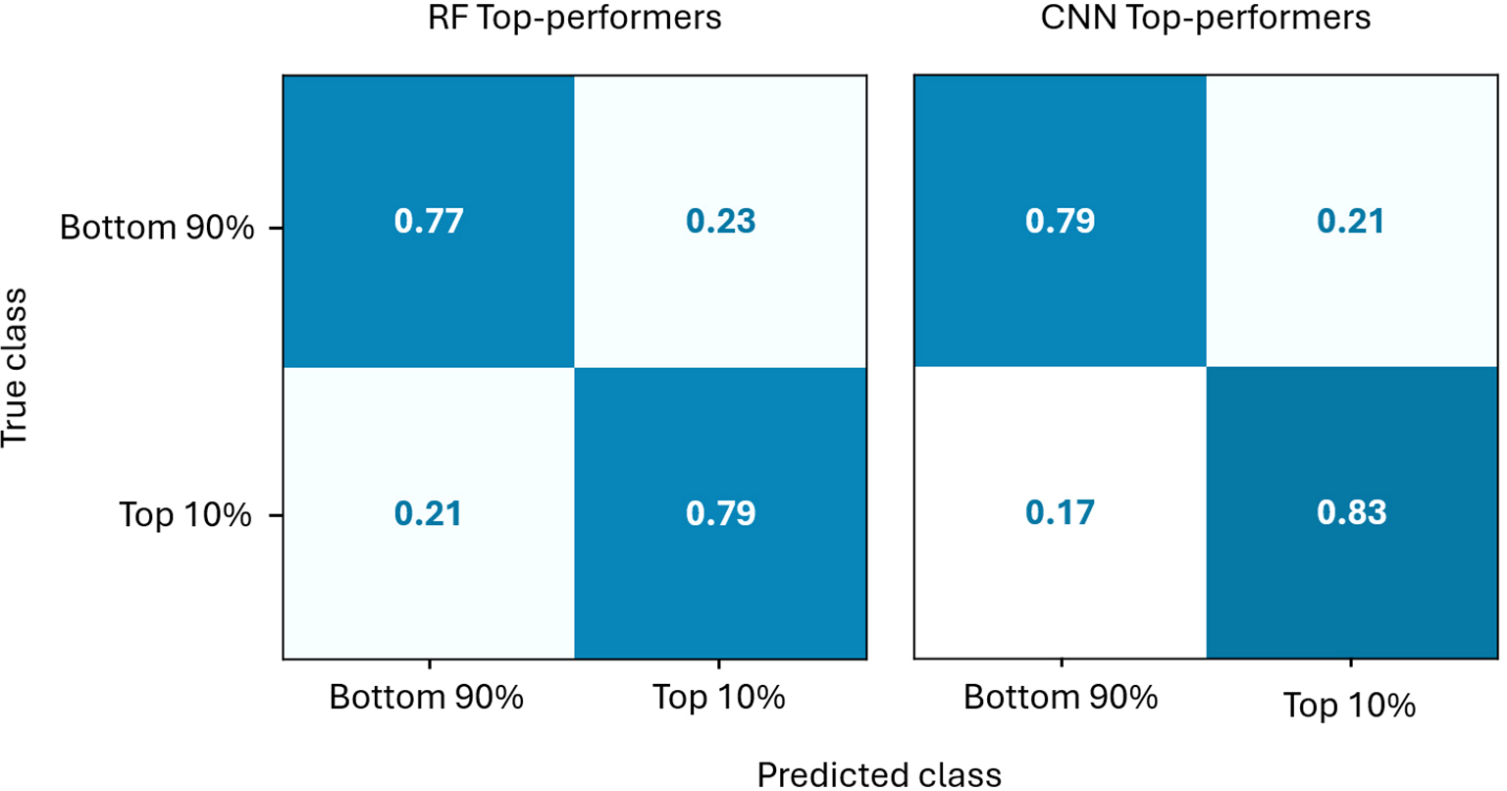
Comparison of the normalised confusion matrices of the CNN and RF Top-performers models on the test set.

The prediction accuracy of the Top-performers models are further detailed in **Figure 9**. The confusion matrices show that both models identify the 10% highest-yielding individuals quite well. Both methods also prioritise minimising false negatives over false positives, thereby reducing the loss of strong candidates. Retaining some lower-potential candidates is less of a concern, as they can be removed in later breeding stages. However, when high-potential candidates are lost, they will likely not be recovered. Furthermore, the CNN model performs slightly better than the RF model, both for the top 10% and the bottom 90% classes, as can be seen in **Figure 9**.

**Figure 10:**
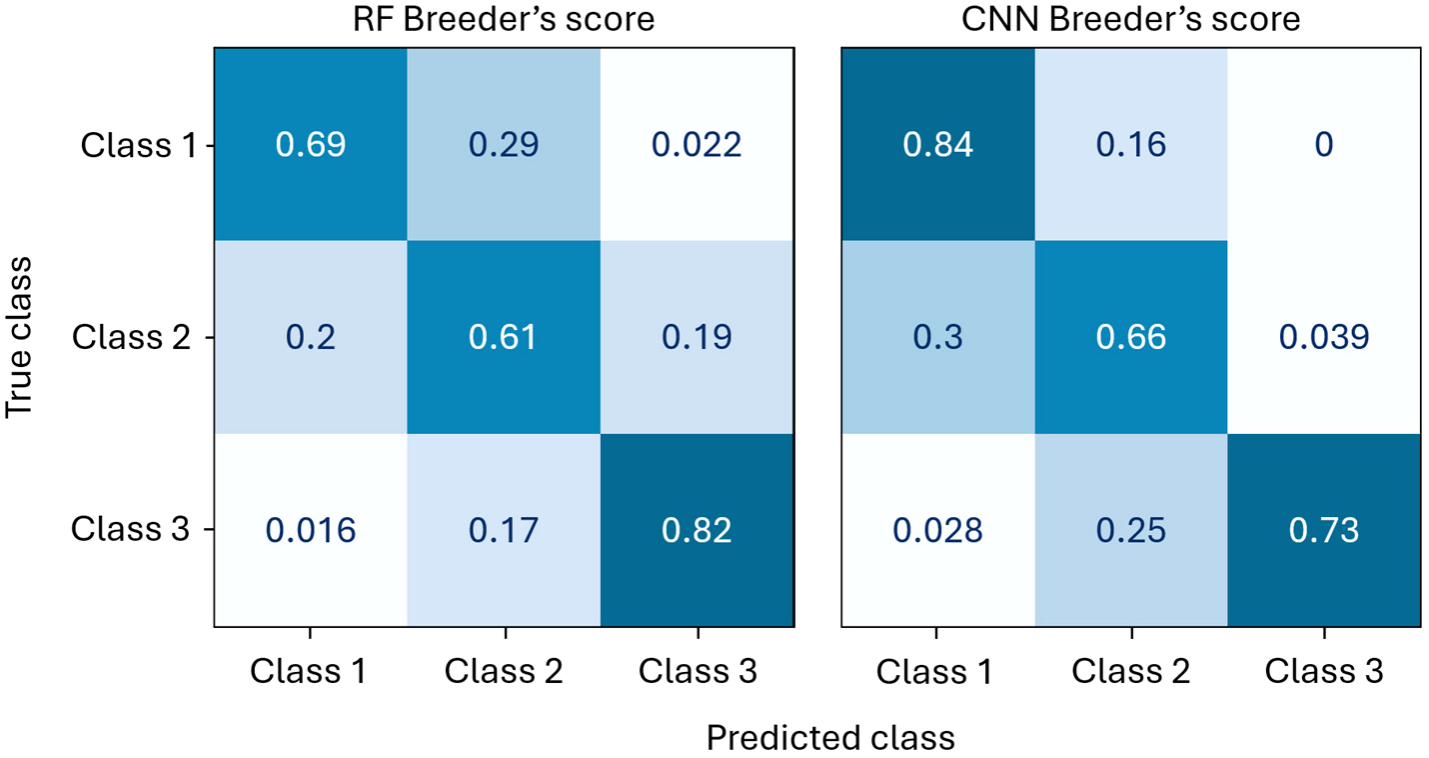
Comparison of the normalised confusion matrices of the CNN and RF Breeder’s score models on the test set.

The confusion matrices of the RF and CNN Breeder’s score models are presented in **Figure 10**. Although the overall performance metrics of the CNN and RF models differ only slightly – no difference in F_1_-measure and only 0.03 in BA (**Table 4**) – their confusion matrices reveal a notable divergence. The CNN method shows superior predictive power for Classes 1 and 2 but performs worse than the RF model for Class 3. Since Class 1 is the most important for the selection process, the CNN approach is preferred.

The confusion matrices also reveal that only a very small number of observations from Class 1 are misclassified as Class 3, and vice versa. These specific misclassifications are likely areas where an ordinal regression model could offer improvements over a classification model. However, given the marginal potential advantage and the added complexity of implementing ordinal regression, it was decided not to pursue this approach further.

## 4 Discussion

### 4.1 Feature importance: insight into the RF models

Feature importance reflects how much a model improves its splitting criterion by partitioning the data according to that feature. This metric is straightforward to estimate in a trained RF, providing valuable insights into the model’s prediction process. While it is also possible to establish feature importance in CNNs, it requires specialised routines that are computationally demanding, making this information less accessible compared to RF models.

**Figure 4** showed that the Top-performers model assigned moderate importance to a larger number of features, while the DMY model identified fewer impactful features, but attributed them considerably higher importance. Because the binary classification task is easier — splitting the data into two classes — many features might contribute to the splits. Each feature that is on average slightly different for Classes 1 and 2 in the training dataset, can make a split that improves the criterion. In contrast, this is harder in the regression model because its task is more complex. Only features with a strong correlation to the outcome will result in substantial criterion enhancement.

**Figure 11:**
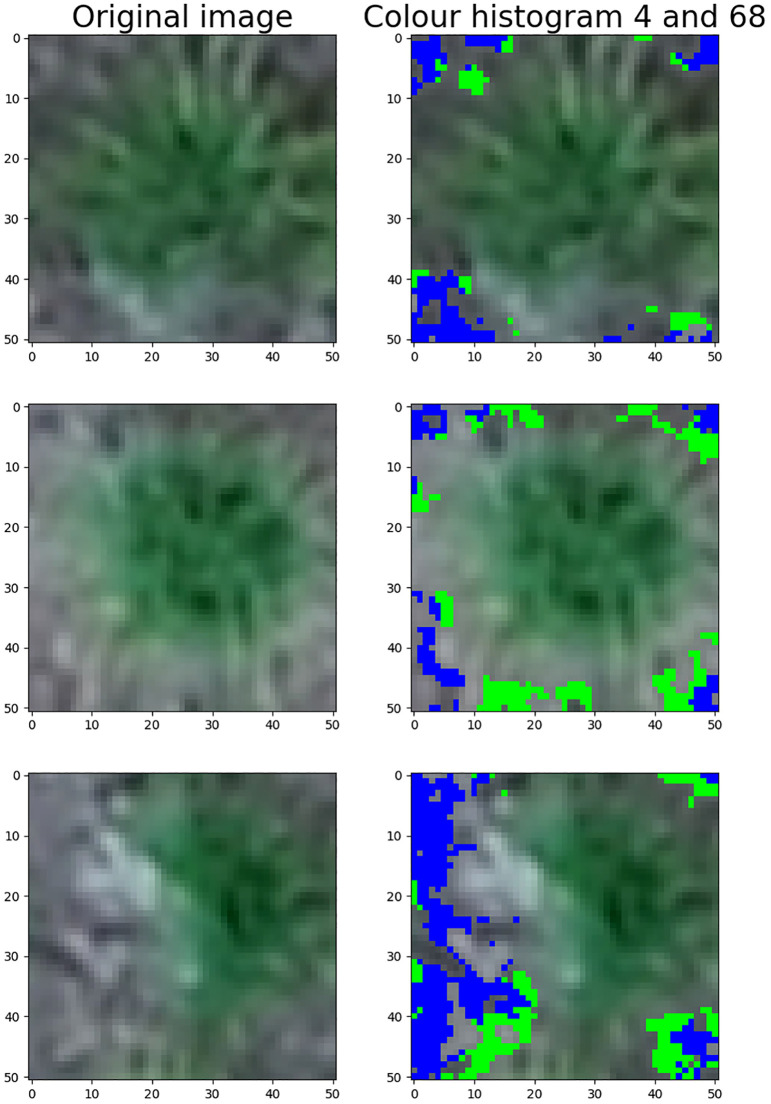
The zones on the image that are captured by features 4 (blue) and 68 (green) of the colour histogram.

The most important features across all models were the two green pixel counters and the 4th and 68th features of the colour histogram. The significance of the green pixels was somewhat expected, as they intuitively correlate with the plant’s biomass. To understand the impact of the colour histogram features, they were highlighted on a few example images in **Figure 11**. Both histogram features seem to capture the intermediately light parts of the soil, which are harder to distinguish from the lighter edges of the plants.

The fifth feature that appeared in the top ten for all three response types was the VARI vegetation index. A study by De Swaef et al. (2021) explored the correlation between the visual breeder’s score and various RGB and thermal-based vegetation indices in different grass species. The VARI was the third highest performing index in this study. The top-performing index was the H (hue) band of the HSV colour space, which aligns with the high importance of the colour histogram features found in this study.

### 4.2 All pre-trained CNN architectures performed similarly

The DenseNet161 architecture without pre-trained weights performed the best overall, although its performance was often very similar to that of the EfficientNet and ResNet50 architectures. On the other hand, VGG-16 performed slightly inferior compared to the other architectures, which is possibly related to its relatively old age. This hypothesis is reinforced by its lower performance in comparable studies (Latif et al., 2022; Castro et al., 2020). However, the Breeder’s score VGG-16 was the only model to exhibit a considerably lower predictive performance. This result can be traced back to the cross-entropy loss function becoming trapped in a local optimum for one of the three splits of the training data. This greatly reduced the average performance and increased the standard deviation of the metrics. Excluding this run reveals an average predictive power that is more similar to that of the other architectures.

Furthermore, the models using the pre-trained parameters demonstrated an inferior prediction performance which was consistent for nearly all architectures and response types. This trend suggests that there was sufficient data to make the optimisation of all parameters to the training dataset advantageous. This is particularly evident in the DMY models, where the contrast between pre-trained and non-pre-trained parameters was most pronounced. Since the original model architectures were designed for classification tasks, a possible explanation is that the regression model deviates the most from the original purpose.

### 4.3 Both CNN and RF models outperform the breeder’s eye

Both the CNN and RF DMY models outperformed the breeder’s score in its ability to predict DMY. These results indicate that the image-based models could not only offer faster, more objective assessments, but could also enhance selection accuracy. This improvement can be attributed to the fact that human assessments are inherently subject to various biases that machine learning models avoid—some potentially useful, such as lowering a score due to disease symptoms, and others less so, such as the influence of previously scored plants, time of day, or the scorer’s state of mind, all of which can reduce accuracy. These advantages of the automated phenotyping approach demonstrate its potential as an alternative to manual phenotyping.

Additionally, the automated method could serve as an initial selection tool rather than a replacement for the breeder’s eye. For example, the Top-performers model can be used to preselect the desired number of high-yielding individuals over multiple cuts, so only this subset has to be evaluated further. The breeder can adjust the DMY threshold to any desired percentage to accommodate his/her objectives and budget. Moreover, the balance between false positives and false negatives can be modified to further optimise how ‘cautious’ the model is in discarding individuals by changing the class weights in the CNN loss function or RF hyperparameters. The model could prioritise the minimisation of false negatives further, ensuring no potentially valuable plants are lost. Even a highly cautious model would considerably reduce the breeder’s workload.

The CNN models consistently demonstrated superior performance compared to the RFs across all response types. CNNs are regarded as the state-of-the-art approach for numerous computer vision tasks, particularly image classification. Therefore, it was not surprising that these models achieved the highest accuracy. Despite the limited size of the dataset, it appears to have provided sufficient information for the CNNs to identify predictive patterns in the images. Additionally, it is plausible that the extracted features for the RF models were suboptimal and the performance could have been improved by more advanced feature engineering. On the other hand, the accuracy of the CNNs did not differ greatly from the RFs. This could be attributed to the limited dataset but also the modest optimisation of the CNN models. Various hyperparameters such as the batch size and the learning rate were not optimised, and only a limited number of architectures were tested.

### 4.4 Comparison to prior studies

The models’ predictive performance and other results obtained in this study were compared to existing research. Several studies have been published that estimate forage yield by means of CNN regression models. Castro et al. (2020); de Oliveira et al. (2021); de Souza Rodrigues et al. (2023) and Oliveira et al. (2022) all achieve comparable results. Castro, de Souza Rodrigues and Oliveira outperform the present study, achieving R² values between 0.75 and 0.79. In contrast, de Oliveira reports slightly lower accuracy, with R² values ranging from 0.38 to 0.62. The better-performing studies have several characteristics in common. Firstly, they use higher-resolution images, with Castro and Souza Rodrigues employing nearly double the resolution used in the present study. Secondly, instead of estimating the yield for individuals, they focus on plots containing multiple plants. This resulted in a more bell-shaped distribution of the measured biomass and fewer outliers, which are favourable properties for predictive modelling. Finally, the models in these studies were trained for a considerably larger number of epochs. While the present research was limited to 15 epochs due to computational constraints, Castro, de Oliveira, and Souza Rodrigues trained their models for 200 - 500 epochs.

Conversely, to the best of our knowledge, there are no other published studies that use a model similar to the Top-performers model. While some studies conducted binary classification of plant images, the use of different modelling techniques, crops and plant traits of interest prevented a direct comparison of the results (Koh et al., 2021; Abdullahi et al., 2017; Momeny et al., 2020; Chipindu et al., 2020). This lack of comparable research confirms the value of the present study in addressing this knowledge gap.

Similarly, no published studies were found that use the breeder’s scores as a response variable, but there are papers employing the same modelling approaches. However, the comparison of results remains difficult, as the classification tasks in these studies focus on diseases and weed species, which are likely easier to visually distinguish than breeder’s scores from 2D images. Furthermore, all studies used images captured with handheld cameras, resulting in considerably higher resolution at the expense of increased time and labour. Nevertheless, it is still interesting to explore these studies due to the technical similarities in their use of CNN models. **Table 5** provides an overview of three comparable studies, detailing their methods and results.

**Table 5:** Comparison of three research papers exploring image-based classification of crops to the results for the Breeder’s score classification model developed in the present study.

Source	Crop	Classification	Best CNN architecture	Number of classes	Size of the dataset	Performance
Latif et al. (2022)	Rice	Disease	Modified VGG-19	6	2,167	*F*_1_: 0.96
Mathulaprangsan et al. (2020)	Rice	Disease	DenseNet161	6	12,223	CA: 0.96
Chen et al. (2022)	Cotton	Weeds	RepVGG-B1	15	5,187	*F*_1_: 0.99
This study	Tall fescue	Yield	DenseNet161	3	4,224	BA: 0.74

CA, Classification Accuracy.

Latif et al. (2022) conducted a study on rice disease classification, encompassing six disease types. They used several pre-trained model architectures, including DenseNet201, VGG-16, and VGG-19. Their comparison of models using pre-trained parameters or newly trained parameters was most interesting for this paper. The majority of models demonstrated improved performance when evaluated with newly trained parameters, which confirms the results obtained in the present study. Mathulaprangsan et al. (2020) used two different ResNet and DenseNet architectures to predict disease classes in rice, of which DenseNet161 performed the best. However, all model architectures showed comparable performance, similar to the findings of the current study. The same observation was made by Chen et al. (2022), who compared 35 different pre-trained architectures to classify weeds in cotton fields. The performance of their highest- and lowest-scoring architectures differed only 4 percentage points.

## 5 Conclusion

The breeder’s eye has proven to be an effective method for plant phenotyping, with successes dating back to the beginning of plant breeding. However, its subjective and time-intensive nature has motivated the search for automated phenotyping approaches. In the present paper, RGB imaging was combined with CNN and RF techniques to assess the phenotype of TF individuals. Three response types were evaluated: a regression model with DMY as the response variable, a binary classification model to identify the top 10% highest yielding individuals and a multi-class classification model to predict the breeder’s score. Additionally, a linear model using the breeder’s score to predict DMY was developed to serve as a benchmark for comparison with the image-based DMY models.

The CNN models slightly outperformed the RFs for all three response types, but both methods clearly surpassed the predictive power of the breeder’s score. Thus, the tested automated phenotyping approach not only offers improvements in cost, efficiency and objectivity, but also enhances selection accuracy. Furthermore, the automated method could complement rather than replace the breeders’ expertise by serving as an initial selection tool, thereby reducing the breeder’s workload while maintaining their crucial role in the process. To conclude, the automated phenotyping approach explored in this study could offer a valuable alternative or addition to traditional visual selection. By accelerating the phenotyping process, it brings resilient and high-yielding varieties one step closer to realisation.

Further research could expand upon this concept by using an average breeder’s score, derived from the evaluation of several breeders, to obtain a more nuanced understanding of ‘the’ breeder’s score. Additionally, using various datasets from different seasons, repeated measurements within seasons, several locations, and various flight times could improve the general applicability of the model. Moreover, due to time constraints, each condition was tested using only three different splits into training and validation sets and finally evaluated on only one test set. Conducting additional splits would have enhanced the reliability of the results and reduced the standard deviation. Also, using cross-validation for these splits would have been preferable, as it ensures the data is systematically partitioned into non-overlapping splits, rather than randomly divided into groups.

## Data Availability

The datasets presented in this study can be found in online repositories. The names of the repository/repositories and accession number(s) can be found below: https://zenodo.org/records/14289667. All scripts used are provided in the following GitHub repository: https://github.com/SarahGhysels/Estimation-of-individual-plant-performance-in-tall-fescue-through-RGB-image-analysis.
